# The major histocompatibility complex homozygous inbred Babraham pig as a resource for veterinary and translational medicine

**DOI:** 10.1111/tan.13281

**Published:** 2018-06-01

**Authors:** J. C. Schwartz, J. D. Hemmink, S. P. Graham, E. Tchilian, B. Charleston, S. E. Hammer, C.‐S. Ho, J. A. Hammond

**Affiliations:** ^1^ The Pirbright Institute Pirbright Surrey UK; ^2^ The Roslin Institute, Royal (Dick) School of Veterinary Studies University of Edinburgh Midlothian UK; ^3^ Livestock Genetics The International Livestock Research Institute Nairobi Kenya; ^4^ Institute of Immunology, Department of Pathobiology University of Veterinary Medicine Vienna Vienna Austria; ^5^ Gift of Life Michigan Ann Arbor Michigan

**Keywords:** genotyping, SLA, SSP typing, Sus scrofa, swine leukocyte antigen

## Abstract

The Babraham pig is a highly inbred breed first developed in the United Kingdom approximately 50 years ago. Previous reports indicate a very high degree of homozygosity across the genome, including the major histocompatibility complex (MHC) region, but confirmation of homozygosity at the specific MHC loci was lacking. Using both direct sequencing and PCR‐based sequence‐specific typing, we confirm that Babraham pigs are essentially homozygous at their MHC loci and formalise their MHC haplotype as Hp‐55.6. This enhances the utility of the Babraham pig as a useful biomedical model for studies in which controlling for genetic variation is important.

1

Pigs are a fundamental food producing animal and important biomedical model. The consumption of pork continues to rise in both the developed and developing world, particularly in Asia,^1^ and preventing and controlling infectious disease remains a priority.^2^, ^3^ Reduced disease burden enables increases in farming density and outputs, improves animal welfare and can reduce the chance of zoonotic disease transmission. Healthier animals can also significantly improve the health and livelihood of small scale and subsistence producers. In addition, the similarities in physiology and organ size mean that pigs are an excellent disease model with the potential to provide organs for human transplantation.^4^, ^5^ To enable future disease, vaccine and translational research, a more detailed understanding of the genetic variation that underpins differential immune responses in pigs is essential.

Domesticated pigs have maintained a significant level of genetic diversity, both within and between breeds, despite strong selection for production traits and inbreeding.^6^ Inevitably, this diversity correlates with significant variation at the polymorphic immune loci. For the pig major histocompatibility complex (MHC, also referred to as swine leukocyte antigen [SLA]), there are currently 238 MHC class I alleles and 223 MHC class II alleles described for *Sus scrofa* in the Immuno‐Polymorphism Database (IPD)‐MHC database (http://www.ebi.ac.uk/ipd/mhc/group/SLA).^7^ The antibody lambda locus also appears to be highly polymorphic, even among commercial pigs with a similar genetic background,^8^ and the T‐cell loci appear variable in gene content, at least between breeds (Schwartz, J.C., T. Connelley, and J.A. Hammond, unpublished). This diversity is problematic for infectious disease research and quantitative trait mapping studies in which complex and uncontrolled genetic variation may confound results and reduce statistical power. Immunogenetic variation also presents significant problems for preclinical studies with the pig as a model and future efforts to enable xenotransplantation. For example, porcine endogenous retroviruses (PERVs), encoded in the pig genome, have impeded organ xenotransplantation as they pose a risk if passed to humans. These elements have recently been removed using genome editing in an outbred pig.^9^ The same work on a large inbred pig (that can produce suitably sized organs) would decrease the possibility of additional uncharacterized PERVs that are likely in outbred populations. Furthermore, having a defined MHC allows one to predict tolerance and associated clinical interventions. For instance, the power of the inbred Babraham pig model has recently been shown in a genome‐wide analysis which identified minor histocompatibility antigens involved in corneal transplant rejection.^10^ This work was only possible using individuals with a controlled genetic background and a defined MHC region in order to facilitate matching and mismatching of histocompatibility loci. Large inbred pig models are therefore fundamentally important scientific resources.

In the United Kingdom, and likely the whole world, the Babraham pig is the only extant example of a large inbred pig breed. While there are several MHC inbred miniature pig breeds that have been developed, including the NIH and Yucatan miniature pigs,^11^, ^12^ these are less representative of commercial breeds. As a consequence, the Babraham pig has great potential to play an important role in studying infectious diseases in pigs and as a preclinical model for human disease. Babraham pigs were derived from a Large White commercial background by Dr Richard Binns at the Babraham Institute (UK) during the 1970s.^13^ Multiple skin grafts were performed across potential parents and selective breeding was carried out between those individuals in which least cross‐rejection was observed. This was continued for five generations, rejecting defective individuals and those with residual skin graft rejection, and which produced individuals that tolerated skin grafts. This tolerance indicated functional homozygosity at least for the MHC antigens and probably also for a high proportion of minor histocompatibility loci. After 20 generations, a restriction fragment length polymorphism study showed a level of inbreeding homozygosity comparable to inbred strains of mice.^13^ During the period from this 1999 study until 2016, there were approximately 15 generations and recovery from a bottleneck of nine sows and three boars with the current population originating from five sows and one boar (animal records from The Pirbright Institute). At this point, single nucleotide polymorphism (SNP) analysis using the Illumina PorcineSNP60 chip suggested approximately 85% homozygosity across the Babraham genome, based on 59 852 genotyped SNPs.^10^ Other animals with the same level of inbreeding and homozygosity are not available for large veterinary species.

The SNP chip analysis by Nicholls et al^10^ indicated complete homozygosity across the MHC region of chromosome 7. However, measuring MHC variation using commercial SNP assays is not always accurate as much of the variation over polymorphic loci falls below the minimum minor allele frequency to be included in the assay. In addition, structurally variable haplotypes often confound the mapping of second‐generation sequencing technologies that produce relatively short reads used to design the SNP chip. Indeed, using SNPchimp^14^ to search the Illumina 60 k SNP array showed only two SNPs present in the vicinity of the classical MHC class I loci (ie, within the interval of Sscrofa10.2, chr 7:24 600 000‐24 765 000). Confirming that the Babraham MHC is homozygous would add significant value to this animal line as both a veterinary and biomedical model. We therefore used two different typing methods to confirm MHC homozygosity and formalise the Babraham MHC haplotype.

The genes targeted for cDNA sequencing were the classical MHC class I genes *SLA‐1*, *SLA‐2* and *SLA‐3*, the non‐classical class I genes *SLA‐6*, *SLA‐7* and *SLA‐8*, and the class II genes *SLA‐DQA*, *SLA‐DQB1* and *SLA‐DRB1*. All known SLA alleles within the IPD‐MHC database were downloaded and used for oligonucleotide primer design (Table 1). Total RNA was extracted from peripheral blood mononuclear cells derived from six animals (as distantly related as possible and including non‐breeding individuals) using TRIzol (Thermo Fisher Scientific, Loughborough, United Kingdom) following manufacturer's instructions. Complementary DNA (cDNA) was generated using the Superscript III reverse transcriptase kit (Thermo Fisher Scientific) following manufacturer's instructions. Polymerase chain reaction (PCR) amplicons were generated from this cDNA, ligated into pGEM‐T Easy vector (Promega, Madison, Wisconsin), and transformed into NEB 5‐alpha chemically‐competent *Escherichia coli* (New England Biolabs, Ipswich, Massachusetts). Approximately 584 individual clones were selected by positive colony PCR result and submitted to Source BioScience (UK) for sequencing. Sanger chain‐termination sequencing was performed using either of the vector‐specific T7 (forward) or SP6 (reverse) primers. The chromatograms from the individual sequencing reads were then compared with the known alleles within the IPD‐MHC database.

**Table 1:** Oligonucleotide primers used for amplification of SLA genes

Gene	Orientation	Sequence (5′‐)	CDNA position	Domain
SLA‐1,‐2, ‐3, ‐7, and ‐8	Sense	GACACGCAGTTCGTGHGGTTC	153‐163	α1
SLA‐6	Sense	AGGACCCGCGTCTGGAGAAG	150	α1
SLA‐1,‐2,‐3,‐6, ‐7, and ‐8	Anti‐sense	CTGGAAGGTCCCATCCCCTG	789‐799	α3
SLA‐1,‐2,‐3, ‐6, and ‐7	Anti‐sense	GCTGCACMTGGCAGGTGTAGC	851‐861	α3
SLA‐DQA	Sense	GAGCGCCTGTGGAGGTGAAG	54	Leader
SLA‐DQA	Sense	GACCATGTTGCCTCCTATGGC	85	α1
SLA‐DQA	Anti‐sense	CAGATGAGGGTGTTGGGCTGAC	398	α2
SLA‐DQA	Anti‐sense	GACAGAGTGCCCGTTCTTCAAC	462	α2
SLA‐DQB1	Sense	GAGACTCTCCACAGGATTTCGTG	98	β1
SLA‐DQB1	Anti‐sense	ACTGTAGGTTGCACTCGCCG	395	β2
SLA‐DRB1	Sense	GGGACAYCSCACMGCATTTC	89	β1
SLA‐DRB1	Sense	GAGTGYCRTTTCTTCAVYGGGAC	127	β1
SLA‐DRB1	Anti‐sense	CAGAGCAGACCAGGAGGTTGTG	421	β2
SLA‐DRB1	Anti‐sense	GGTCCAGTCTCCATTAGGGATC	552	β2

Abbreviation: SLA, swine leukocyte antigen.

To further confirm SLA homozygosity in the Babraham pigs, genotyping of *SLA‐1*, *SLA‐2*, *SLA‐3*, *DRB1*, *DQB1* and *DQA* was performed on the genomic DNA from 22 animals (including the six animals used for cDNA analysis) using PCR‐based assays with sequence‐specific typing primers (PCR‐SSP) as previously described.^15^, ^16^ The typing primer panel has since been modified to accommodate for the increasing number of SLA alleles and allele groups (details not shown).

Both SSP typing and sequencing methods confirmed homozygosity at the *SLA‐1*, *SLA‐2*, *SLA‐DQA*, *SLA‐DRB1* and *SLA‐DQB1* loci (Table 2). The sequenced region of *SLA‐DQB1*, containing the majority of both beta domains, could not differentiate between alleles SLA‐DQB1*08:01 and SLA‐DQB1*08:02, which differ from each other at two nucleotide positions outside of the sequenced region (ie, at positions +52 and +606). This gene was nevertheless identical over the sequenced region in all animals based on reads from eight clones per animal. Only three sequencing reads from two animals were recovered for *SLA‐3*. One of these reads corresponded with SLA‐3*04:02 and the remaining two reads corresponded with SLA‐3*04:03, indicating that at least one of the six animals is a heterozygote at this locus. These two alleles differ only in the alpha‐3 domain, by both a 12‐bp insertion in SLA‐3*04:03 and a single non‐synonymous mutation 9 bp upstream of the insertion. However, it is uncertain what, if any, influence these differences have on peptide‐binding and receptor interactions, especially as this region is distal from the peptide‐binding regions of the alpha‐1 and alpha‐2 domains. The paucity of *SLA‐3* reads is likely due to the co‐amplification of *SLA‐3* cDNA with *SLA‐1* and *SLA‐2*, both of which are considered more highly expressed.^17^, ^18^ The haplotype that corresponds to the genotype SLA‐1*14:02‐SLA‐3*04:03‐SLA‐2*11:04‐DRB1*05:01‐DQB1*08:01 has been previously designated by the ISAG/IUIS‐VIC SLA Nomenclature Committee Hp‐55.6. The class I haplotype Hp‐55.0 was originally described in the ESK‐4 cell line,^19^ while the class II haplotype Hp‐0.6 has been detected in several pig breeds including Yucatan,^20^ Austrian Pietrain,^21^ Chinese Bama miniature pigs,^22^ as well as the SK‐RST cell line.^19^

**Table 2:** Classical MHC class I and class II genotypes of inbred Babraham pigs

	SLA‐1	SLA‐2	SLA‐3	DRB1	DQA	DQB1
Sequencing	*14:02 a	*11:04 b	*04:03 c/*04:02	*05:01	*01:03	*08:01 or *08:02
SSP typing	*14:02	*11:04	*04:XX	*05:XX	*01:XX	*08:XX

Abbreviations: MHC, major histocompatibility complex; SLA, swine leukocyte antigen; SSP, sequence‐specific typing primers.

a Previously known as SLA‐1*es11.

b Previously known as SLA‐2*es22.

c Previously known as SLA‐3*04es32.

Sequencing reads were additionally obtained for the non‐classical MHC class I genes (*SLA‐6*, *SLA‐7*, and *SLA‐8*) due to broad primer specificity. A total of six identical reads from three animals were identified for *SLA‐6*, all of which contained the intron between the first two alpha domains, and thus originated from either unspliced mRNA or contaminating genomic DNA. Despite this, both exons were in frame and putatively functional. All of these reads also differed by at least 4 bp from the nine known *SLA‐6* alleles in IPD‐MHC, with five alleles being equally close (SLA‐6*01:01, SLA‐6*03:01, SLA‐6*04:01, SLA‐6*05:01 and SLA‐6*06:01). Reads specific for *SLA‐7* (*n* = 1) and *SLA‐8* (*n* = 8, from four animals) were also detected, likely due to the degenerate nature of the *SLA‐6* primers used for cDNA amplification. Only three alleles of *SLA‐7* are currently described within the IPD‐MHC database, and the closest of these, SLA‐7*01:01, differs by 2 bp to the single read sequenced from the Babraham samples. For *SLA‐8*, all of the sequencing reads were identical to each other and were also exact matches for known alleles SLA‐8*01:01, SLA‐8*04:01 and SLA‐8*05:01. As the sequenced reads did not span the entire transcript, it could not be ascertained which, if any, of these alleles correspond to the Babraham *SLA‐8*. Thus, the sequencing results suggested that the Babraham animals were identical to each other for at least the *SLA‐6* and *SLA‐8* non‐classical MHC class I loci, while only a single read was obtained for *SLA‐7*.

For inclusion into the IPD‐MHC database, the Babraham‐derived alleles presented here have been deposited into GenBank (accessions: MH107868—MH107877). This study shows that the inbred Babraham pigs are functionally MHC homozygous. Taken together with the high level of inbreeding as measured by SNPs over the entire genome,^10^ this confirms the Babraham pig as a very valuable model for swine and human disease research, as well in wider biomedical applications. This value can only increase as our ability to edit mammalian genomes and produce gene‐edited animals improves.
